# Evaluation of the association between TNF-α-1031 T/C polymorphism with oral lichen planus disease

**DOI:** 10.1186/s12903-024-03939-x

**Published:** 2024-02-05

**Authors:** Mohammad Hesam Marabi, Hamid Reza Mozaffari, Haniyeh Ghasemi, Masoud Hatami, Kheirollah Yari

**Affiliations:** 1 Student Research Committee, School of Dentistry, Kermanshah University of Medical Sciences, Kermanshah, Iran; 2 Medical Biology Research Center, Health Technology Institute, Kermanshah University of Medical Sciences, Kermanshah, Iran; 3 Department of Oral and Maxillofacial Medicine, School of Dentistry, Kermanshah University of Medical Sciences, Kermanshah, Iran

**Keywords:** Oral lichen planus, Tumor necrosis factor-α, Genetic polymorphism, PCR-CTPP, SNP

## Abstract

**Background:**

Oral lichen planus (OLP) is a T-cell-mediated autoimmune disease that affects the epithelial cells of the oral cavity. This study was performed to investigate any possible relationship between − 1031(T/C) polymorphism (rs1799964) of the tumor necrosis factor α (TNF-α) gene with the risk and severity of oral lichen planus (OLP) disease among an Iranian population.

**Method:**

Saliva samples were collected from 100 patients with OLP and a similar number of healthy controls (age and sex-matched). Then, DNA was extracted from the collected samples for genotyping TNF-α-1031 T/C polymorphism using the PCR-CTPP method. The results were assessed using SPSS software.

**Results:**

The findings revealed a significantly higher prevalence of the C allele in OLP patients (53%) compared to healthy controls (36%), suggesting an association between TNF-alpha gene polymorphism and OLP. A multivariate logistic regression analysis supported this finding, as the presence of the C allele was significantly associated with an increased risk of OLP [χ2 = 4.17, *p* = 0.04, 95% CI = 1.01–2.65, OR = 1.64]. However, our data indicated no significant association between TNF-alpha-1031 T/C gene polymorphism and OLP severity.

**Conclusions:**

These findings provide the first evidence supporting a possible role of TNF-α-1031 T/C gene polymorphism in OLP susceptibility in the Iranian population. The findings of this study demonstrate a positive association between TNF-α-1031 C/T allele distribution and the risk of OLP disease in the Iranian population. Therefore, carrying the C allele may increase the susceptibility to OLP disease.

**Supplementary Information:**

The online version contains supplementary material available at 10.1186/s12903-024-03939-x.

## Background

Lichen planus is a common chronic inflammatory mucosal and skin disorder that affects approximately 0.5–4% of the worldwide population, with a malignant transformation rate of 1.4% [1, 2]. Also, OLP had a higher prevalence among middle-aged females [3, 4], while it is also rare in children (0.03%) [5]. Patients often are unaware of their oral condition [6]. Compared to the cutaneous form of this disease, oral involvement is more frequent and treatment-resistant [7]. Oral lichen planus (OLP) forms white lines or plaques, erythema, erosion, or blisters that mainly affect the buccal mucosa, tongue, and gums. Lesions are usually bilateral and often appear as a combination of clinical subtypes [8]. Clinically, OLP contains various forms, including reticular, erosive, papular, atrophic, plaque-like, and bullous presentations [9]. Reticular and erosive forms are the most common types of oral lichen planus [10]. According to the studies, OLP could increase the risk of oral squamous cell carcinoma, which is an oral potentially malignant disorder [11, 12]. A more significant malignant potential has been identified for the atrophic and erosive forms of OLP [6]. The etiology of OLP disease is still debated. However, the immune-response pathogenesis of this disease has been recognized [5]. Some studies have indicated intrinsic and extrinsic factors such as dental materials, some medications, stress, liver diseases, hepatitis C virus infection, tobacco, alcohol consumption, and genetic factors that could be involved in OLP susceptibility [6, 13, 14]. Substantial evidence suggests that OLP is triggered by an antigen that alters keratinocyte antigen expression or unmasking of an antigen, making them susceptible to cell-mediated autoimmune reactions [15]. Following, it induces the migration of T lymphocytes (mostly CD8+, and some CD4 + cells) into the epithelium and the production of T helper 1 (Th1) cytokines, such as interleukin-2, interferon-gamma and tumor necrosis factor [16], which determine the keratinocytes apoptosis, mucosal basement membrane destruction and long-term chronicity of the disease [15, 17]. Cytokines, such as tumor necrosis factor-α (TNF-α), a pro-inflammatory cytokine and potent immune system inducer, are thought to play a pivotal role in the development of OLP and other autoimmune and inflammatory diseases [18]. Elevated serum TNF-α levels have been observed in OLP patients compared to healthy controls [11]. So far, several single nucleotide polymorphisms (SNPs) within the promoter sequence (-238, -244, -308, -376, -489, -575, -610, -851, -857, -863, -1031) of the TNF-α gene have been reported that affect transcriptional regulation and therefore susceptibility of different diseases [19, 20]. The SNP that occurs in the regulatory position − 1031, the promoter of the TNF-α gene, is characterized by the substitution of cytosine for thymine [21]. This polymorphism change could be related to the up-regulation of TNF-α and cause its increase in blood circulation [19, 21–23]. No study has investigated the association of TNF-α -1031 polymorphism with OLP susceptibility and severity in an Iranian population. Therefore, our study aims to address this knowledge gap and explore the potential link between TNF-α gene − 1031 T/C polymorphism and OLP in the Iranian population.

## Methods

### Sample collection and ethics

Ethical approval was obtained from the Medical Ethics Committee of Kermanshah University of Medical Sciences with the IRB number of IR.KUMS.REC.1400.424. All participants or their legal guardians were freely asked to sign a consent form to join this study before their enrollment after a clear explanation of the aims and methods of the study according to their knowledge. All the authors adhered to the 1975 Helsinki Declaration and its subsequent revisions.

The sample size of ~ 93 was calculated for each group with an alpha value of 0.05 and a beta value of 20%, but to ensure sample adequacy, 100 subjects were enrolled in each of the case and control groups [24]. The OLP diagnosis was based on the definition of OLP by the World Health Organization [25] and confirmed by pathology reports and clinical manifestations detected by an oral medicine specialist. Inclusion criteria for OLP patients were as follows: 1; diagnosis according to the definition of OLP by the World Health Organization 2; did not receive any relevant treatment before inclusion in this study [24]. The exclusion criteria were defined as any malignancy or autoimmune/inflammatory diseases and systemic disorders. Also, patients with amalgam lichenoid reaction lesions were excluded. A family history of OLP was not considered in the selection of subjects.

### Genetic analysis

Three milliliters of saliva samples were collected from each subject in falcon tubes and immediately transferred to the laboratory on ice for storage at -80^o^C. Genomic DNA was extracted from the saliva samples using the standard (DNrich saliva kit, Cat. No. AESDX1010). Polymerase chain reaction with confronting two-pair primers (PCR-CTPP) technique (using two pairs of primers) was employed to determine the genotype and investigate the TNF-α-1031T/C polymorphism. PCR amplified TNF-a gene with the following primers:

F1: 5′ AAGGCTCTGAAAGCCAGCTG,

R1: 5′ CCAGACCCTGACTTTTCCTTCA,

F2: 5′ GAAGCAAAGGAGAAGCTGAGAAGAC,

R2: 5′ CTTCCATAG CCCTGGACATTCT [26].

The PCR parameters were: one cycle at 95 °C for 10 min, followed by 30 cycles of 95 °C for 1 min, 66 °C for 1 min, 72 °C for 1 min, and a final at 72 °C for 5 min.

Genotypes were identified based on the electrophoresis pattern on a 2% agarose gel.

**Fig. 1:**
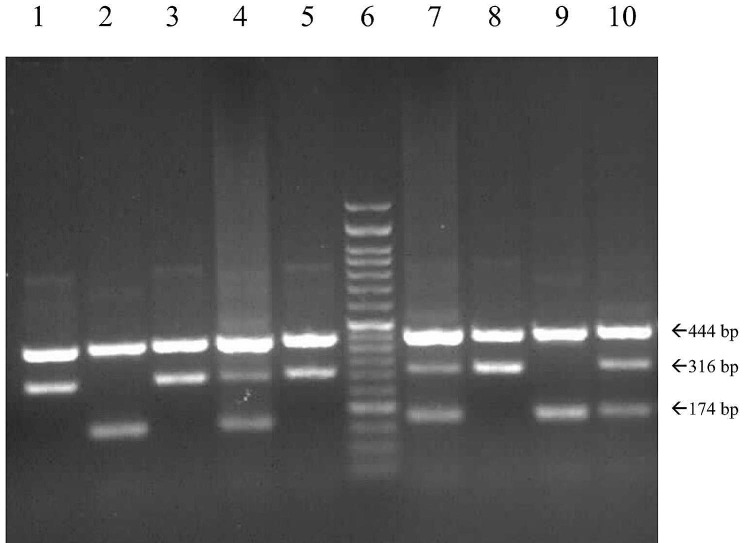
Electrophoresis pattern of PCR-CTPP products for TNF-α-1031 T/C genotypes. Lanes 1, 3, 5, and 8: sample with T/T genotype; Lanes 2 and 9: sample with C/C genotype; Lanes 4, 7, and 10: sample with T/C genotype. Lane 6 DNA size marker (50 bp). (See full, un-cropped version of this image on supplementary Figure S1)

In the obtained electrophoresis pattern, the CC genotype was represented by two bands of 174, 444 bp, the wild genotype (TT) was represented by two bands of 316 and 444 bp, and TC genotype was represented by three bands of 444, 316, and 174 bp (Fig. 1).

### Statistical analysis

The frequency of genotypes under different genetic models and allele distribution between case and control groups were analyzed using the chi-square (χ2) test in Statistical Package for the Social Sciences version 16.0 (SPSS Inc. IBM, New York, USA). A logistic regression model was employed to evaluate the risk of TNF-α-1031 in OLP patients. Observed results were expressed as odds ratios (OR) with 95% confidence intervals. A *p*-value less than 0.05 was considered statistically significant.

## Results

A total of 200 individuals participated in this study, comprising 100 patients (29 males and 71 females, mean age of 48 years) with OLP and 100 healthy controls(28 males and 72 females, mean age of 49 years) matched in terms of sex and age. The highest incidence of OLP was investigated in subjects aged 46–65 years (48%), followed in order of frequency among others aged 19–30 (9%), 31–45 (36%), and > 66 (7%). Also, 65% and 35% of the patients with OLP were diagnosed with clinical erosive and non-erosive subtypes, respectively. An analysis of genotype frequencies indicated no deviation from Hardy–Weinberg equilibrium in the patients (χ^2^ = 0.25, *p* = 0.88) and controls (χ^2^ = 0.026, *p* = 0.98).

The frequency of genotypes and allele − 1031-TNF-α among the studied groups have been shown in Table 1. The frequency of T/T, T/C, and C/C genotypes at position -1031 of TNF-α gene in the patient group were 55%, 37%, and 8%, respectively, and in the control group, they were 67%, 30%, and 3%, respectively. The frequency of 1031-TNF-α mutant genotype (CC) in the patient group was higher than compared to controls (8% and 3%, respectively). However, statistical analysis showed no significant differences in -1031-TNF-α genotypes between the control and patient groups (χ2 = 4.18, *p* = 0.12). Also, genotype frequencies in co-dominant, dominant over-, dominant, and recessive genetic models were not statistically different (*p* > 0.05). The genotype distributions were in Hardy-Weinberg equilibrium in OLP patients and the control group. The analyses revealed a statistically significant difference in allele frequencies between OLP patients and healthy controls (χ2 = 4.17, *p* = 0.042, 95% CI = 1.01–2.65, OR = 1.64). Our result indicates that the presence of the C allele significantly increases the risk of OLP (1.64-fold). Also, comparing genotype and allele frequencies of -1031-TNF-α between erosive and non-erosive clinical subtypes have been presented in Table 2. As indicated in Table 2, no significant association was observed between the TNF-α -1031 T/C allele and genotype in different genetic models with OLP severity (*p* > 0.05).

**Table 1:** Frequency distribution of SNP-1031 genotypes and related alleles of the TNF-α gene in OLP patients and control group

SNP-1031	Patient *N* = 100		Control *N* = 100		χ2	*p* value	OR
**Genotypes**							
Co dominant				4.18		0.12	
TT (Wild)	55		67				
CT (Hetrozygote)	37		30				
CC (Mutant)	8		3				
Dominant				3.02		0.08	
TT	55		67				
C/T + C/C	45		33				
Recessive							
CC	8		3	2.40		0.12	
TT + CT	92		97				
Over-dominant							
CT	37		30	1.1		0.29	
T/T + C/C	63		70				
**Allele**							
T		147	164				
C		53	36	4.17	**0.042**		95% CI = 1.01–2.65, OR = 1.64

**Table 2:** Frequencies of TNF-α gene, SNP-1031 in erosive and non-erosive subtypes

TNF-1031	Erosive group *n* = 65	Non-erosive group *n* = 35	χ2	*p*-value
**Genotypes** Co-dominant				
TT	34 (52%)	21 (60%)	2.07	0.35
CT	27 (42%)	10 (29%)		
CC	4 (6%)	4 (11%)		
Dominant				
TT	34 (52%)	21 (60%)		
CT + CC	31 (48%)	14 (40%)	0.54	0.46
Recessive				
CC	4 (6%)	4 (11%)		
CT + TT	61 (94%)	31 (89%)	0.86	0.35
Over Dominant				
CT	27 (42%)	10 (29%)		
TT + CC	38 (58%)	25 (71%)	1.64	0.2
**Allele**				
T C	95 (73%) 35 (27%)	52 (74%) 18 (26%)	0.03	0.85

## Discussion

Cytokines are considered essential mediators for the development/pathogenesis of inflammatory diseases such as OLP. Regarding this issue, it has been shown that polymorphisms of cytokine genes such as *IFN-γ, TNF-α, TNF-β, IL-4, IL-8*, and *IL-10* are associated with OLP susceptibility [27, 28]. Previously, we reported the genotype and allele frequencies of IL-8) + 781 C/T (in OLP patients among an Iranian population [29], indicating that IL-8 + 781 C/T polymorphisms might be correlated to the severity of OLP. TNF-α gene is a pro-inflammatory and immunomodulator cytokine playing a pivotal role in OLP pathogenesis. The expression of the TNF-*α* gene is regulated by various SNPs (-238, -244, -308, -376, -489, -575, -610, -851, -857, -863, -1031) in the promoter sequence, which consists of 1100 bp of DNA [30]. TNF-α -1031 polymorphism could be related to the up-regulation of TNF-α. Nourian et al. indicated a positive association between polymorphism of − 1031 T > C with the TNF-α mRNA expression level [31]. They demonstrated that the level of the TNF-α gene was higher expressed in genotype TT when compared with the other genotypes. According to our knowledge, this study is the first report on the association of -1031(T/C) polymorphism of the TNF-α gene (rs1799964) with the risk and severity of OLP disease in an Iranian population. Also, this is the first report for evaluating the SNP-1031 as one of the primary SNPs in the regulatory sequence of the TNF-α gene. The findings of this study demonstrated a positive association between TNF-α-1031 C/T allele distribution and the risk of OLP disease in the Iranian population. We collected saliva samples for DNA extraction because it is easy to access, non-invasive, and more practical for evaluation and analysis. Our study by previous reports confirmed that collecting saliva samples is a well-accepted procedure by all individuals [24, 32–34]. Mozaffari et al. reported elevated expression levels of TNF-α in saliva compared with serum samples [33]. They suggest that the measurement of TNF-α in saliva may be a more helpful marker for OLP diagnosis and treatment. Also, Zhang et al. concluded that saliva sampling could be an efficient substitute for serum collection in evaluating pro-inflammatory cytokines in patients with OLP [32]. Ghasemi et al. used saliva samples for genomic DNA extraction and genotyping of SNP IL-8 +781 [24].

Previous studies have demonstrated associations between TNF-α − 1031T/C polymorphism with different oral malignancies and autoimmune or inflammatory diseases such as Behçet’s disease [35], periodontitis [36], Crohn’s disease [37], chronic obstructive pulmonary disease [38], Gastric Carcinoma [39]. These findings suggest that the TNF-α gene may play a significant role in the susceptibility to inflammatory diseases. Majumder et al. concluded that − 1031T/C SNP of the TNF-α gene is associated with increased susceptibility to chronic periodontitis as a common inflammatory oral disease [36]. We concluded that the C allele of -1031 SNP of the TNF-α gene may increase the susceptibility to OLP disease. Our results are in accordance with Akman et al. findings, which showed that in the Turkish population, the TNF-*α*–1031 C allele is associated with susceptibility to Behçet’s disease [35]. Also, in agreement with our results, Sanchez et al. revealed a positive over-representation of homozygous −1031 CC among Crohn’s disease patients of Caucasians of the French-Canadian population.

A systematic review by Zhou and Vieira showed that G/A-308 polymorphism in TNF-α is a potential genetic marker for OLP [40]. Also, Al-mohaya et al. investigated the relationship between TNF-α, TNF-β, and interleukin ten gene polymorphisms in Saudi patients with the risk of OLP. They concluded that polymorphisms of TNF-α (-308G/A), TNF- β + 252 A/G, and IL-10 (-1082G/A, -819 C/T, and − 592 C/A) are associated with sensitivity to OLP [27]. Also, in a study by Kimkong et al., they evaluated the association of three polymorphisms in the promoter region of TNF-α, -863, -308, and − 238 in the Thai population of patients with OLP. In their study, no significant association with the development of OLP was found [41]. Carrozzo et al. analyzed thirteen cytokine genes with 22 single nucleotide polymorphisms. They suggested that the genetic polymorphism of the first intron of the IFN-c promoter gene may be an essential risk factor for developing OLP lesions. At the same time, the increased frequency of the TNF-α-308 allele may contribute to more skin involvement [42]. To our knowledge, this is the first report to study the TNF-α-1031 T/C polymorphism in OLP. As with any other study, this one is not without limitations. Among the limitations of this study were the small sample size of case and control subjects because of the limited available number of patients, lack of evaluation of TNF-α gene expression, and haplotyping with different SNPs.

## Conclusion

The current study investigated the possible relationship between −1031(T/C) polymorphism of the TNF-α gene (rs1799964) and the risk and severity of OLP disease in an Iranian population. Our results indicated a non-significant higher frequency of mutant genotype (CC) in the OLP group compared to the control group. We found that the presence of the C allele significantly increased the risk of OLP disease by 1.64-fold. The results of this study demonstrate a positive association between TNF-α-1031 C/T allele distribution and the risk of OLP disease in the Iranian population for the first time. Therefore, our results support the genetic basis of OLP disease. The results of this study suggest that the -1031TNF-α C allele may be a genetic risk factor for OLP in the Iranian population. Therefore, the results of our study can support the genetic basis of OLP disease. However, further research is warranted to confirm these findings and to elucidate the mechanisms by which the -1031TNF-α C allele increases susceptibility to OLP. Additionally, studies with larger sample sizes in more expansive geographical areas and more detailed evaluations of TNF-α gene expression and haplotyping with different SNPs are necessary to gain a better understanding of the role of the -1031TNF-α C allele in OLP pathogenesis.

### Electronic supplementary material

Below is the link to the electronic supplementary material.

**Supplementary Material 1:** Supplementary figure S1 Full, un-cropped electrophoresis pattern of PCR-CTPP products for position -1031 of TNF-α gene shown in Figure 1

## Data Availability

All data generated or analyzed during this study are included in this published article.

## Abbreviations

OLP: Oral lichen planus
PCR-CTPP: Polymerase chain reaction with confronting two-pair primers
TNF-α: Tumor necrosis factor-alpha
IL: Interleukin
SNP: Single nucleotide polymorphism
