# Supplementary figures and images for: Evaluation of the association between TNF-α-1031 T/C polymorphism with oral lichen planus disease
Source: BMC Oral Health. 2024 Feb 5;24:189. doi: 10.1186/s12903-024-03939-x (PMC10845614; doi:10.1186/s12903-024-03939-x)

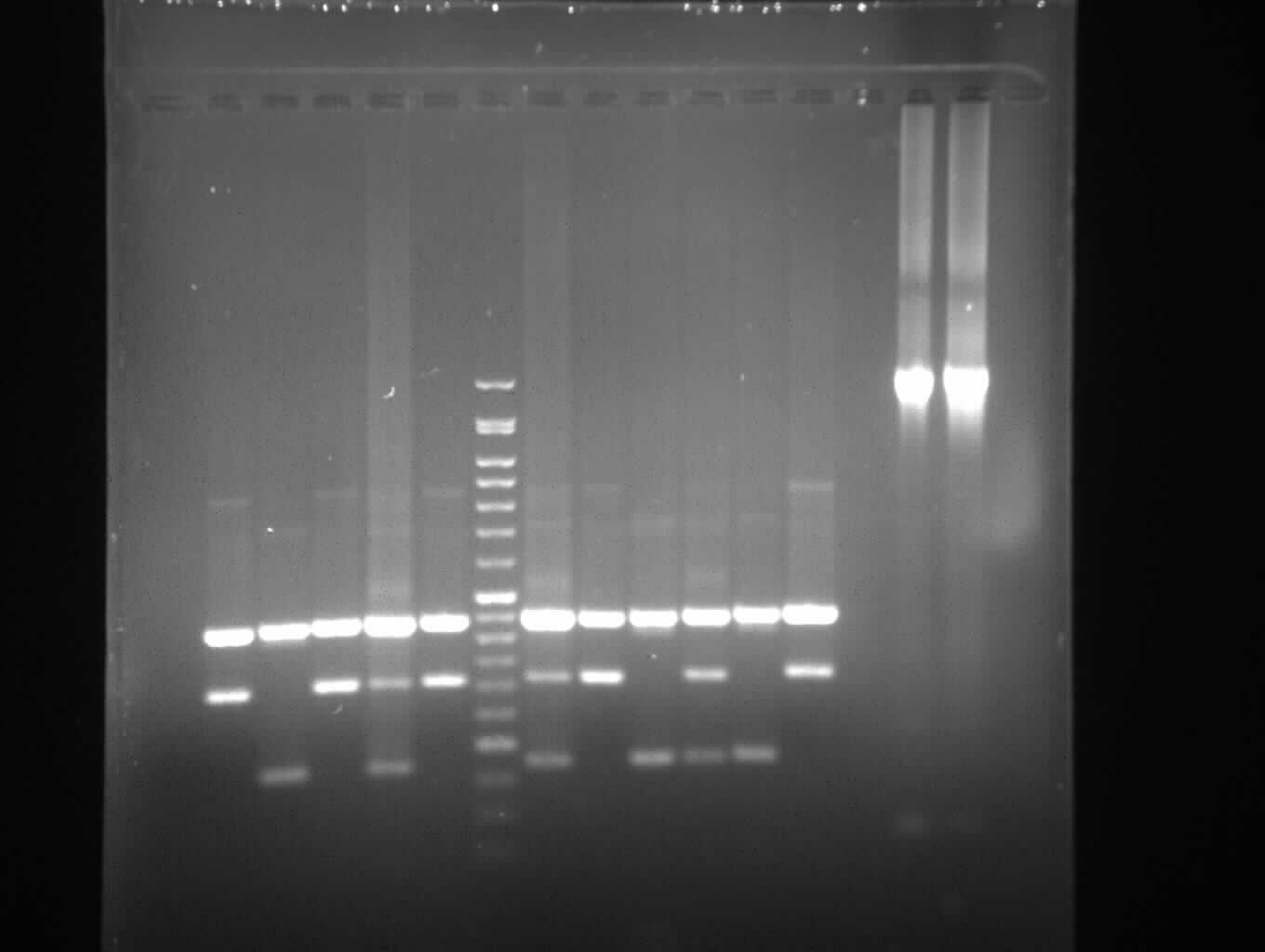

Supplement: Supplementary file 1 — Supplementary Material 1: Supplementary figure S1 Full, un-cropped electrophoresis pattern of PCR-CTPP products for position -1031 of TNF-α gene shown in Figure 1 [file 12903_2024_3939_MOESM1_ESM.jpg]
